# Tau depletion prevents progressive blood-brain barrier damage in a mouse model of tauopathy

**DOI:** 10.1186/s40478-015-0186-2

**Published:** 2015-01-31

**Authors:** Laura J Blair, Haley D Frauen, Bo Zhang, Bryce A Nordhues, Sara Bijan, Yen-Chi Lin, Frank Zamudio, Lidice D Hernandez, Jonathan J Sabbagh, Maj-Linda B Selenica, Chad A Dickey

**Affiliations:** Department of Molecular Medicine, University of South Florida, Byrd Alzheimer’s Institute, 4001 E. Fletcher Ave MDC 36, Tampa, FL 33613 USA; James A. Haley Veteran’s Hospital, 13000 Bruce B. Downs Blvd., Tampa, FL 33612 USA; Department of Pharmaceutical Science, University of South Florida, Byrd Alzheimer’s Institute, 4001 E. Fletcher Ave University of South Florida, Tampa, FL 33613 USA

**Keywords:** rTg4510, Alzheimer’s disease, Blood-brain barrier, Tau, Vascular

## Abstract

**Introduction:**

The blood-brain barrier (BBB) is damaged in tauopathies, including progressive supranuclear palsy (PSP) and Alzheimer’s disease (AD), which is thought to contribute to pathogenesis later in the disease course. In AD, BBB dysfunction has been associated with amyloid beta (Aß) pathology, but the role of tau in this process is not well characterized. Since increased BBB permeability is found in tauopathies without Aß pathology, like PSP, we suspected that tau accumulation alone could not only be sufficient, but even more important than Aß for BBB damage.

**Results:**

Longitudinal evaluation of brain tissue from the tetracycline-regulatable rTg4510 tau transgenic mouse model showed progressive IgG, T cell and red blood cell infiltration. The Evans blue (EB) dye that is excluded from the brain when the BBB is intact also permeated the brains of rTg4510 mice following peripheral administration, indicative of a bonafide BBB defect, but this was only evident later in life. Thus, despite the marked brain atrophy and inflammation that occurs earlier in this model, BBB integrity is maintained. Interestingly, BBB dysfunction emerged at the same time that perivascular tau emerged around major hippocampal blood vessels. However, when tau expression was suppressed using doxycycline, BBB integrity was preserved, suggesting that the BBB can be stabilized in a tauopathic brain by reducing tau levels.

**Conclusions:**

For the first time, these data demonstrate that tau alone can initiate breakdown of the BBB, but the BBB is remarkably resilient, maintaining its integrity in the face of marked brain atrophy, neuroinflammation and toxic tau accumulation. Moreover, the BBB can recover integrity when tau levels are reduced. Thus, late stage interventions targeting tau may slow the vascular contributions to cognitive impairment and dementia that occur in tauopathies.

**Electronic supplementary material:**

The online version of this article (doi:10.1186/s40478-015-0186-2) contains supplementary material, which is available to authorized users.

## Introduction

The blood-brain barrier (BBB) is a physical barrier of endothelial cells, supported by astrocytes, that selectively limits the passage of peripheral blood components and pathogens into the brain, while allowing the passage of essential nutrients [1,2]. In aging, there is a decline in the stability of the BBB leading to increased permeability [3]. This disruption of BBB integrity is exacerbated in Alzheimer’s disease (AD) [4-6], allowing peripheral immune cells access to the brain and perhaps exacerbating pathology by promoting detrimental neuroinflammation [7-10]. Most evidence suggests that this increase in BBB damage is driven by accumulation of amyloid-beta (Aß), particularly along the vasculature [11-13]. However, the microtubule associated protein tau has been shown to accumulate as puncta in perivascular spaces in sporadic AD with cerebral amyloid angiopathy (CAA) [14]. BBB damage is also observed in tauopathies that lack Aß over-production [15-17], suggesting a role for tau in BBB damage [18-21], although this has yet to be proven.

We turned to the well-characterized tetracycline inducible rTg4510 mouse model to address the gaps in our knowledge about the role of tau in BBB damage. These mice exhibit a robust pathological profile, which includes tau tangle formation, neuroinflammation, neuronal loss and cognitive deficits [22-24]. Overall tau accumulation is detectable by 1 month of age, while insoluble tau, neurotoxicity, and atrophy, appear as early as 2.5 months of age [22]. These pathologies progressively worsen and by 8.5 months of age, roughly 80% of neurons in the CA1 and dentate gyrus are lost [22,24-27]. Importantly, tau expression can be suppressed by doxycycline (DOX) administration in this model, a feature that can be exploited to determine whether pathophysiological consequences of tau expression can be halted. For example, tau suppression was previously found to rescue memory deficits and neuronal loss in this model, without affecting the accumulation of neurofibrillary tangles [23].

Thus, we used this model to determine if and when BBB integrity might be compromised, and whether this was caused by tau accumulation alone, by brain volume loss, or by some other pathology yet to be described. We also exploited the DOX-regulation of this model to determine if BBB damage was permanent, and whether specific features of the model were most closely linked to BBB dysfunction. We found that tau accumulation does eventually lead to BBB disruption, but there is an extremely high threshold of neuronal loss that the BBB can withstand before losing integrity. In fact, onset of BBB disruption was concomitant with the appearance of perivascular tau, suggesting a relationship between these two pathologies. BBB function was largely restored when tau was suppressed, even well after onset of BBB dysfunction. These data suggest that tau accumulation can disrupt BBB integrity and that BBB dysfunction coincides with the appearance of perivascular tau. These findings are important considerations for drug discovery efforts based on this model because they suggest that the vasculature can recover when tau levels are reduced, suggesting that late stage interventions targeting tau may be useful for slowing the vascular contributions to cognitive impairment and dementia that occur in tauopathies.

## Materials and methods

### Study approval

All applicable international, national, and/or institutional guidelines for the care and use of animals were followed. All animal handling and procedures were carried out in accordance with the University of South Florida’s Institutional Animal Care and Use Committee (IACUC) in accordance with the Association for Assessment and Accreditation of Laboratory Animal Care International (AALAC) regulations.

### Mouse colony and tissue processing

The rTg4510 colony was bred and maintained as previously described [22,23]. Mice were harvested at 1-, 3-, 6-, 9-, and 12-months old (N = 5-8 per genotype). At sacrifice, mice were perfused with 0.9% saline solution; brains were rapidly removed and fixed with 4% paraformaldehyde overnight. Following sucrose gradients up to 30%, tissue was sectioned using a sliding microtome at a thickness of 25 μm.

### Doxycycline treatment procedure

In the DOX treated groups, 10.5- to 11-month old rTg4510 (N = 7) and wild-type (N = 7) mice were treated for ~5 weeks with DOX and harvested at 12-months old. When DOX treatment was started, mice were given 1.5 g/L DOX (Sigma Cat# 9891, St. Louis, MO;) in 4% sucrose water for 48 hours. After two days, DOX water was replaced by tap water and mice were fed a DOX diet containing 0.2 g/kg DOX (Harlan Cat# TD.00502, Indianapolis, IN) until sacrifice.

### Intracardiac injection of Evans blue (EB)

Intracardiac injection of EB (Sigma) was performed on 6- (N = 7), 9- (N = 8), 12- (N = 8), and 12-month old DOX-treated (N = 7) rTg4510 and wild-type littermates as previously described [28], with slight modification. Mice were anesthetized by inhalation of precisely 2% isoflurane, to prevent variance of BBB opening due to isoflurane inhalation [29]. While in dorsal recumbency, hair was removed from the injection site, the site was sterilized, and a percutaneous stick was made through the rib cage between the 4^th^ and 5^th^ intercostal space from the mouse’s left side. 20 mg/kg EB in sterile PBS was injected into the left ventricle at a rate of approximately 200 μL/min forty minutes before sacrifice. Tissue was harvested as describe above, including transcardiac perfusion to remove all blood from blood vessels.

### Immunohistochemistry and immunofluorescence

Tissue was stained free floating as previously described [30] with minor modifications using antibodies directed against tau (H-150) (Santa Cruz, Dallas, Tx; 1:5000), heat shock protein 27 kDa (Hsp27; c-20) (Santa Cruz; 1:5000), Glial fibrillary acidic protein (GFAP; DAKO, Carpinteria, CA; 1:3000), CD3^+^ (AbD serotec, Raleigh, NC; 1:30,000), and CD4^+^ (AbD serotec; 1:30,000) with swine anti-goat IgG BIOT (1:200), goat anti-rabbit IgG BIOT (1:10,000), goat anti-mouse IgG BIOT (1:3000) and goat anti-rat IgG BIOT (1:1000) secondary antibodies (Southern Biotech, Birmingham, AL). Blood vessels were stained using DyLight 488 labeled Lycopersicon Esculetum (Tomato) Lectin (Vector Laboratories; Burlingame CA; 1:200). Fluorescently stained tissues were stained free-floating as previously described [31]. Briefly, following permeabilization, tissue was incubated with tau (H-150; 1:1000) and/or Hsp27 (1:100) primary antibody overnight. Following PBS washes, tissue was incubated with Alexa Fluor 488 donkey anti-goat IgG (Invitrogen, Grand Island, NY; 1:1000) and/or Alexa Fluor 633 goat anti-rabbit IgG (Invitrogen; 1:1000) secondary antibody for 2 hours, followed by an overnight incubation with Tomato Lectin 488 (1:500). Brightfield tissue was mounted, dehydrated, and coverslipped using DPX (VWR, Atlanta, GA). Fluorescently stained tissue was mounted and then coverslipped using ProLong Gold Antifade Reagent (Invitrogen). Hematoxylin (Sigma) and eosin (Sigma) (H&E) staining was performed on mounted tissue as previously described [32] to view red blood cells (RBCs). Following nuclear and cytoplasmic staining, tissue was rapidly dehydrated in ethanol gradients, cleared in xylenes, and coverslipped using DPX.

### Tissue imaging and quantification

Stained tissue was imaged using a Mirax slide scanner. IgG, tau (H-150), Hsp27, and GFAP were quantified in the cortex and hippocampus by segmentation of positive areas using NearCYTE software. As previously reported, this software allows for the segmentation of positive staining based on hue, saturation, and color intensity compared to that of the background in very specific regions of the tissue [33,34]. Consistent color settings were used to analyze each piece of tissue, which was selected for regions of interest based on a mouse brain atlas, across each stain. CD3^+^ and CD4^+^ T cells along with H&E positive red blood cells were manually counted by a blinded investigator throughout each section.

For Lectin/tau staining images, z-stack images were taken each 1 μm through the tissue using Leica TCS SP2 Confocal Microscope equipped with a 63x/ 1.4-0.60 PLAN APO Oil objective using Argon and Red HeNe lasers and I3 and N2.1 filters respectively. EB and Hsp27/tau stain was imaged with an AxioCamMR3 camera on a Zeiss AxioImage.Z1 using a 10×/0.25 dry Zeiss EC Plan-Neofluar 10×/0.30 M27 objective. Positive signal was excited using an EXFO X-Cite fluorescence illuminator with 43HE: Cy3 filter (excitation 550/emission 605) at a set exposure time of 750 ms for all tissue. Analysis of EB images was performed using ImageJ software (National Institutes of Health). Images were converted to 8-bit and a threshold of 100-255 was applied. Particle analysis was performed on areas between 0 and infinity pixels^2^ and a circularity of 0.50-1.00, which allowed for the analysis of all positive EB staining.

### Image analysis and statistics

Six to eight sections per mouse were averaged together as a single representative value for a brain region. Statistical analysis was performed either using a 1-way or 2-way analysis of variance (ANOVA) (Age and Genotype) or (Age and Treatment) as appropriate. Values compared between genotypes of a single age group were analyzed by t-test. Values were considered significant when *p* <0.05. Graphs were generated using GraphPad Prism 5.0.

## Results

### IgG extravasation in aged rTg4510 mice

**Figure 1:**
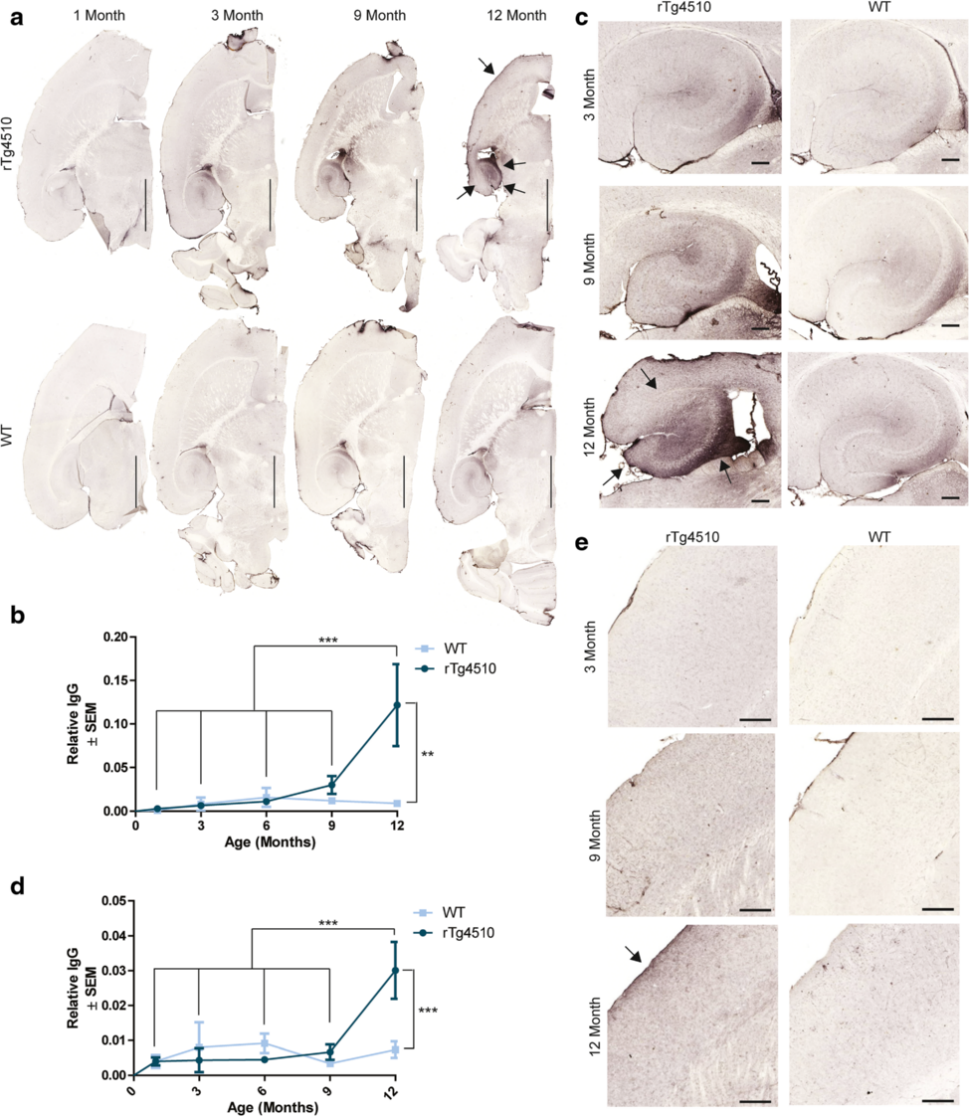
**IgG accumulates with age in the rTg4510 mouse model. a** Goat anti-mouse IgG staining on tissue from 1-, 3-, 9-, and 12-month old rTg4510 and wild-type (WT) mice. Arrows indicate areas of most intense IgG accumulation in the frontal cortex and hippocampus. Scale bar represents 2000 μm. Quantification of the relative area ratio of IgG accumulation in the **b** hippocampus (± SEM). ** *p* <0.01, ***p < 0.001. 5× magnification of the **c** hippocampus of 3-, 9-, and 12-month old rTg4510 and wild-type mice showing progressive accumulation of IgG in the hippocampus with age. Arrow indicates CA3 area has more IgG accumulation. Scale bar represents 200 μm. Quantification of the relative area ratio of IgG accumulation in the **d** frontal cortex (± SEM) of rTg4510 compared to wild-type mice with age. ***p < 0.001. 5x magnification of the **e** frontal cortex of 3-, 9-, and 12-month old rTg4510 and wild-type mice showing progressive accumulation of IgG in the cortex with age. Arrow highlights the most accumulation of IgG is on the surface of the cortex. Scale bar represents 200 μm.

The first indication that there could be BBB damage in the rTg4510 mouse model was the presence of intense background staining when using anti-mouse IgG secondary antibodies on aged tissue, similar to what has previously been shown following AAV delivery of P301L tau in wild-type mice [19,20]. This anti-IgG reactivity was only observed in aged rTg4510 and not in age-matched wild-type littermates or younger rTg4510 mice. To investigate the progression and severity of the IgG infiltration, a more thorough examination of IgG immunoreactivity was then performed on rTg4510 and wild-type mice at 1-, 3-, 6-, 9-, and 12-months old. We found that rTg4510 mice displayed progressively higher visible levels of anti-mouse IgG immunoreactivity than age-matched wild-type littermate (Figure 1a). The most visibly apparent regions affected by IgG accumulation were the hippocampus, stemming from the fimbria of the hippocampus, and the frontal cortex, most notably along the edges of the tissue. Both the hippocampus and frontal cortex have been characterized to have extensive tau accumulation and severe neuron loss in rTg4510 model [24]. In fact, the hippocampus has been shown in wild-type rodents to be susceptible to BBB impairment [35,36], so this natural predisposition combined with the intense pathology which is found throughout the hippocampus made this region particularly interesting. Anti-mouse IgG quantification in the hippocampus revealed a significant increase in 12-month old rTg4510 mice (Figure 1b) compared to age-matched wild-type mice. This same phenomenon was mirrored in previous studies using hippocampal tissue of AD cases [5]. Upon closer visual examination of the hippocampus in rTg4510 mice, the highest accumulation of IgG was found in the CA3 (Figure 1c), however the dentate gyrus and the CA1 region were also noticeably darker than the wild-type littermates. Although a similar pattern of staining were seen in the hippocampus of the wild-type mice, the overall staining was much lighter than that found in the rTg4510 model. Since regions adjacent to periventricular areas are most prone to BBB permeability, it was not surprising the CA3 region had the most IgG immunoreactivity [37]. In the frontal cortex, another region that has significant tau accumulation and atrophy [38], IgG immunoreactivity was also significantly increased in 12-month old rTg4510 mice compared to wild-type littermates (Figure 1d and e). This immunoreactivity was most marked on the edge of the tissue with a gradient to lighter IgG reactivity radiating laterally inward. Based on these findings, we speculated aging, combined with chronic tau overexpression, could lead to BBB disruption.

### Extravasation of immunoglobulin and EB in 12-month old rTg4510 mice

**Figure 2:**
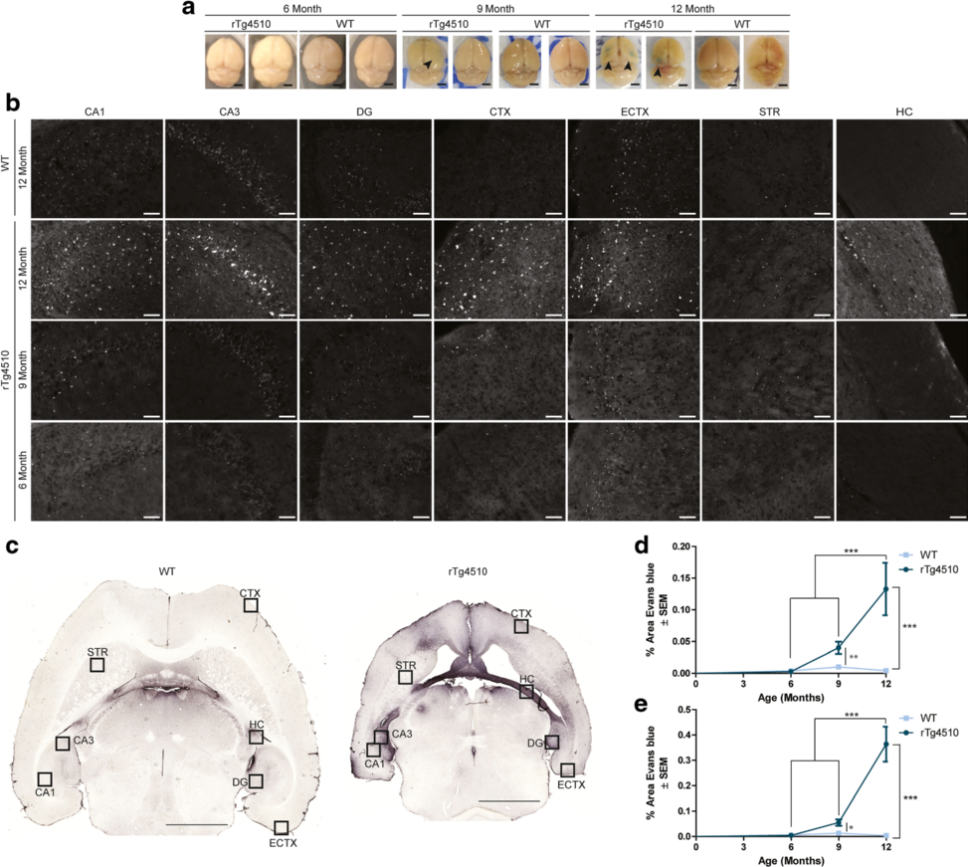
**Evans blue (EB) extravasation reveals blood-brain barrier dysfunction in rTg4510 mice by 12 months of age.** Representative images of whole brains immediately following perfusion displayed gross **a** EB extravasation (*black arrowhead*) in 9- and 12-month old rTg4510 mice. Scale bar represents 200 μm. Representative 20x images of **b** EB fluorescence in the CA1, CA3, dentate gyrus (DG), frontal cortex (CTX), entorhinal cortex (ECTX), striatum (STR), and hippocampal commissure (HC) of 12-month old wild-type and 6-, 9-, and 12-month old rTg4510 mice. Scale bar represents 50 μm. Representative whole brain image stained with **c** anti-mouse IgG from 12-month oldwild-type and rTg4510 mice showing map of locations used for EB fluorescent imaging in **b**. Scale bar represents 2000 um. Quantification of EB fluorescence in the **d** hippocampus (± SEM) of 6-, 9-, and 12-month old rTg4510 and wild-type mice. ***p <0.001, ** *p* <0.01. Quantification of EB fluorescence in the frontal cortex (± SEM) **e** of 6-, 9-, and 12-month old rTg4510 and wild-type mice. *** *p* <0.001, ** *p* <0.05.

To more thoroughly investigate BBB permeability in these mice, three cohorts of rTg4510 mice and wild-type littermates aged 6-, 9-, and 12-months old were intracardially injected with EB, a dye that is typically occluded from the brain except when the BBB is damaged. EB is commonly used for BBB studies and emits fluorescence when bound to albumin in the blood [39-41]. Mice were perfused to remove all blood from the brain. Thus any fluorescent signal in the brain would indicate EB bound to albumin in the parenchyma and not the surrounding vasculature, indicative of BBB breakdown. Whole brains from mice that received intracardiac EB injections were imaged following sacrifice (Figure 2a). Blue staining was visible in each of the 12-month old rTg4510 tau mice predominantly in the cortical layers superior to the hippocampus, similar to the pattern of accumulation of IgG on the surface of the cortex (Figure 1e). This blue staining was not visible in 6-month old rTg4510 mice or 12-month old wild-type littermates, but was seen to a lesser extent in some 9-month old rTg4510 mice.

Brain tissue was fixed, sectioned, and mounted for brain region specific analysis of EB. EB fluorescence was found to be highest in 12-month old rTg4510 mice, compared to younger rTg4510 mice or age-matched wild-type mice (Figure 2b). Regions with high EB expression corresponded to those with greatest anti-mouse IgG immunoreactivity (Figure 2c), the highest being the hippocampus and the cortex, followed by the entorhinal cortex and hippocampal commissure. This again shows that periventricular regions are most susceptible to BBB dysfunction [37], and that this is severely exacerbated in the rTg4510 mouse model. Thus, we analyzed the extravasation of EB in the hippocampus and frontal cortex, since these areas showed prominent IgG infiltration. Significant EB fluorescence was observed in the hippocampus (Figure 2d) and frontal cortex (Figure 2e) of 12-month old rTg4510 mice but not in 6-month old rTg4510 mice or any wild-type mice. Similar to IgG, extravasation of EB was greatest in the CA3 region of the hippocampus (Additional file 1: Figure S1), compared to the CA1 and dentate gyrus, however both of these regions still showed significant EB accumulation. 9-month old rTg4510 mice showed high levels of EB fluorescence in both the hippocampus and frontal cortex which were significant by Student t-test, but not ANOVA. Thus rTg4510 mice can develop a major BBB defect, but this is only significant after 9 months of age, well after robust brain atrophy and tau accumulation begins in this model.

### Increasing tau accumulation is accompanied by glial activation

**Figure 3:**
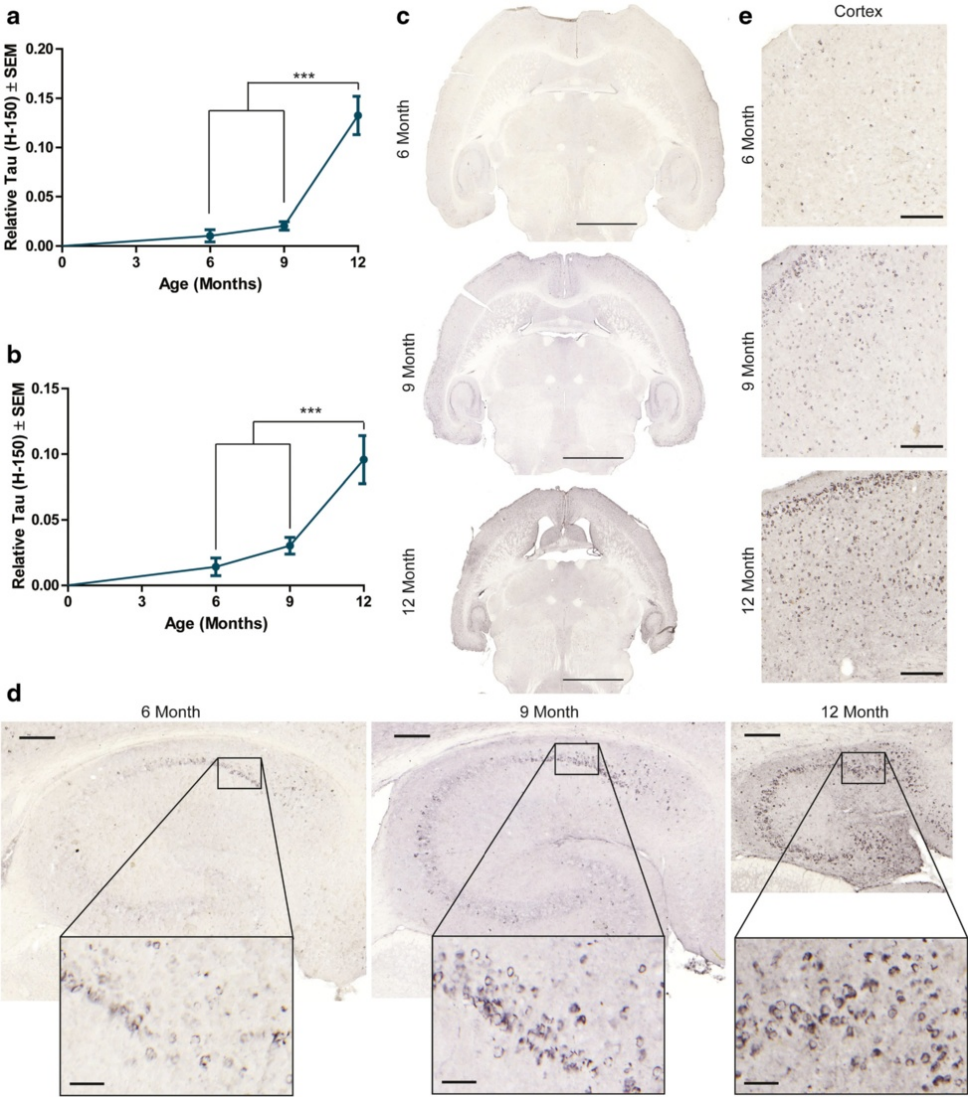
**Tau (H-150) accumulation in aging rTg4510 mice.** Tissue from aging rTg4510 mice were stained with total tau (H-150) and quantified for total tau (H-150) in the **a** hippocampus (± SEM) and **b** frontal cortex (± SEM) in rTg4510 mice. *** *p* <0.001. Representative images of a whole section stained with **c** total (H-150) tau from a 6-, 9-, and 12-month old rTg4510 mouse. Scale bar represents 2000 μm. Representative images from corresponding **d** hippocampus and **e** frontal cortex are shown. Scale bars represent 200 μm; hippocampus inset scale bar represents 50 μm.

As previously reported [22,23,42], tau accumulation was significantly increased in an age-dependent manner in the hippocampus (Figure 3a) and frontal cortex (Figure 3b) of rTg4510 mice. Tau accumulation throughout the brain accompanied by visible brain atrophy, including the hippocampus (Additional file 1: Figure S2), was progressively increased in mice from 6 to 12 months of age (Figure 3c). Tau staining in the hippocampus (Figure 3d) and frontal cortex (Figure 3e) showed region-specific increases in tau accumulation with age.

**Figure 4:**
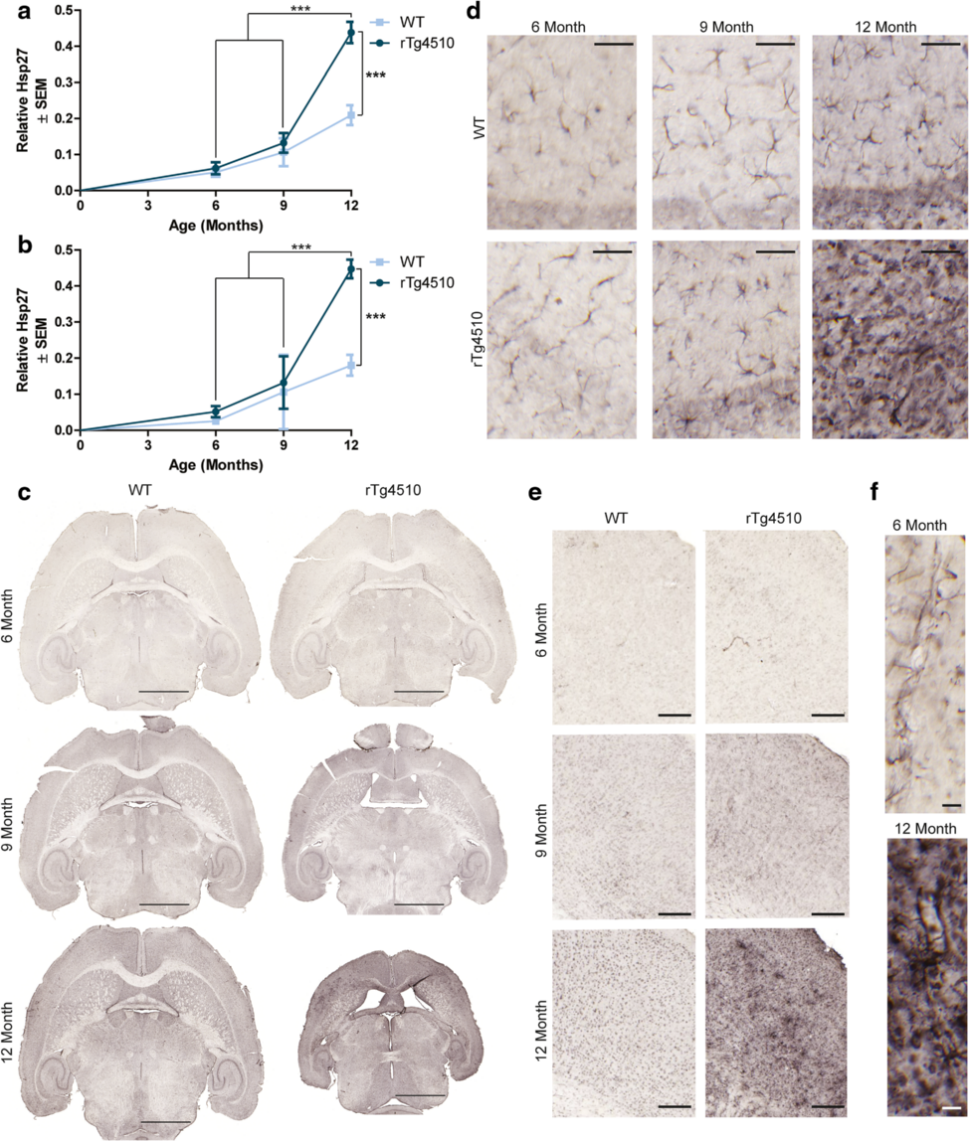
**Hsp27 abundance increases with age in rTg4510 mouse brain.** Hsp27 staining was quantified in the **a** hippocampus (± SEM) and **b** frontal cortex (± SEM) of 6-, 9-, and 12-month old rTg4510 and wild-type mice. ***p <0.001. Representative images of the **c** whole brain, **d** hippocampus (from CA1 region), and **e** frontal cortex are shown. Scale bars represent 2000 μm, 50 μm, and 200 μm respectively. High magnification images of **f** blood vessels in the hippocampus from 6- and 12-month old rTg4510 mice. Scale bar represents 20 μm.

It is well-documented that tau accumulation in the rTg4510 mouse model induces neuroinflammation, including microgliosis and astrocytosis [24,30,32,43]. In fact, there is a robust inflammatory response that begins somewhere between 2 and 4 months of age in these mice [43]. This inflammation is closely correlated with neurodegeneration in this model and can be reversed by DOX-treatment [20,38,43]. Here we focused on astrocytosis rather than microgliosis given the intimate role of astrocytes with BBB stability [44]. We investigated the levels and morphology of heat shock protein 27 kDa (Hsp27) in 6-, 9-, and 12-month old rTg4510 and age-matched wild-type mice. Hsp27 was selected because it has been shown to increase with age [22], is highly expressed in activated astrocytes [45], is found lining blood vessels [46], is constitutively expressed in vascular endothelial cells [47], and has been implicated in regulation of the BBB through its role in actin stabilization [48]. We found that Hsp27 levels in the hippocampus (Figure 4a) and frontal cortex (Figure 4b) were significantly increased in rTg4510 mice at 12 months of age. This progressive increase was clearly visible throughout entire brain sections (Figure 4c). Upon closer examination of the hippocampus (Figure 4d) and frontal cortex (Figure 4e), Hsp27 immunoreactivity appeared adjacent to blood vessels and with age these astrocytes appeared more activated (Figure 4f). The age-related increases in Hsp27 were significantly exacerbated by tau overexpression (Figure 4a and b).

**Figure 5:**
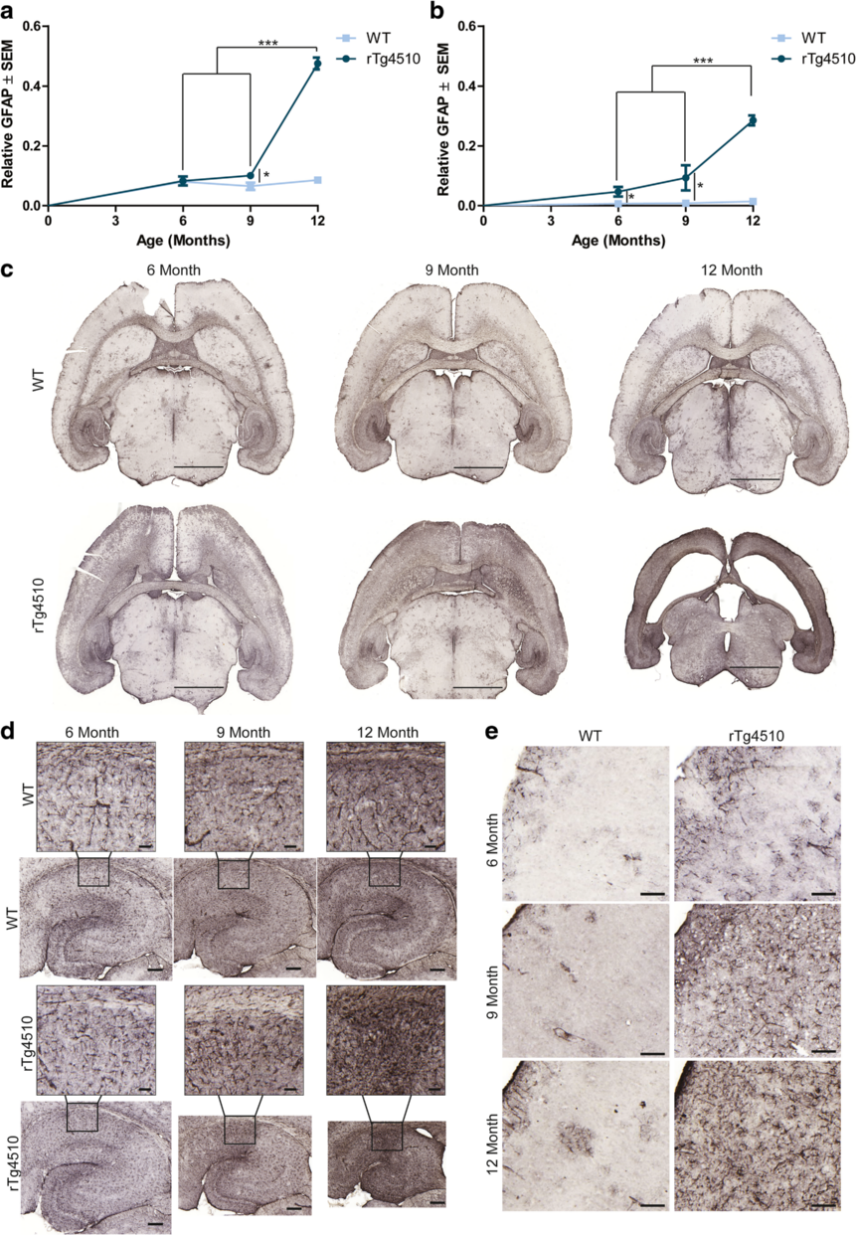
**GFAP positive astrocytes accumulate with age in rTg4510 mice.** Area ratio positive for GFAP immunoreactivity was quantified in the **a** hippocampus (± SEM) (*p = 0.01696; ***p <0.001) and **b** frontal cortex (± SEM) (6-month *p = 0.020102, 9 month *p = 0.04813, ***p <0.001) of wild-type and rTg4510 mice at 6, 9 and 12 months of age; **c** representative whole brain images are shown. Scale bar represents 2000 μm. Representative images of **d** hippocampus (± SEM) and **e** cortex (± SEM) of GFAP immunostained tissue from 6-, 9- and 12-month old wild-type and rTg4510 mice. Scale bar represents 100 μm; inset scale represents 20 μm.

To confirm the accumulation of astrocytes as detected by Hsp27 staining, we also stained tissue from 6-, 9-, and 12-month old rTg4510 and wild-type mice for GFAP, a more traditional marker of astrocytes [18,49,50]. Similar to the results from Hsp27, GFAP was significantly elevated in the hippocampus (Figure 5a) and frontal cortex (Figure 5b) of aged rTg4510 mice compared to wild-type controls. Whole brain sections show that accumulation of GFAP followed a similar pattern to that of tau in the rTg4510 mice (Figure 5c). Closer examination of the hippocampus (Figure 5d) showed that there was an age related increase in GFAP immunoreactivity that was exacerbated in the rTg4510 mice starting at 9 months of age, significantly higher than wild-type mice. While the rTg4510 cortex (Figure 5e) showed less of an age-related increase in GFAP immunoreactivity, there was still a clear elevation starting at 6 months of age that progressively increased.

### Invasion of red blood cells and T cell lymphocytes is evident in aged rTg4510 mice

**Figure 6:**
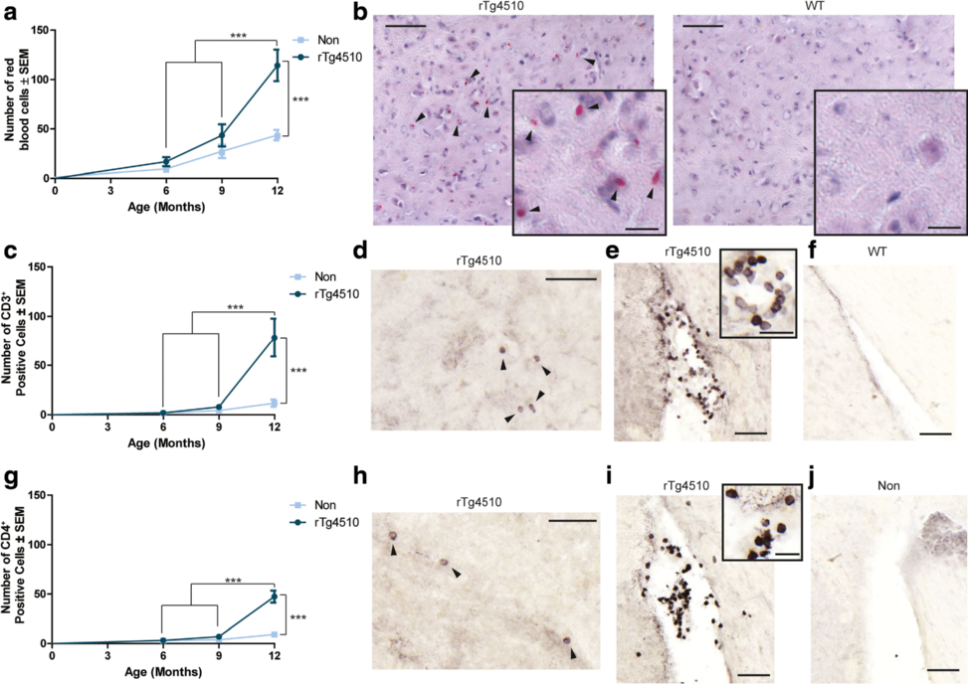
**Erythrocytes and leukocytes infiltrate the brain of aged rTg4510 mice.** Sections from 6-, 9-, and 12-month old rTg4510 and wild-type mice were stained with hematoxylin and eosin (H&E) and **a** red blood cells (RBCs) (± SEM) were manually counted throughout the brain. *** *p* <0.001. A 40x representative image of **b** H&E staining from 12-month old rTg4510 and wild-type mice is shown. Scale bar represents 50 μm; 20 μm inset. Arrowheads indicate positive RBCs. T cell lymphocytes were stained with **c** CD3^+^ antibody and counted (± SEM) in 6-, 9-, and 12-month old rTg4510 and age-matched wild-type mouse tissue. *** *p* <0.001. CD3^+^ T cells were found in 12-month old rTg4510 mice in the **d** hippocampus and in and around the **e** ventricles**.** Arrowheads indicate positive CD3^+^ T cells. However, there was very little positive staining was found in the **f** tissue or ventricles of age-matched wild-type littermates. Scale bar represents 50 μm in the hippocampus and ventricle images; inset scale bar represents 20 μm. T cell lymphocytes stained for **g** CD4^+^ were counted (± SEM) in 6-, 9-, and 12-month old rTg4510 and wild-type mouse tissue. *** *p* <0.001. CD4^+^ T cells were found in the **h** hippocampus and in and around the **i** ventricles of 12-month old rTg4510 backspace. Similar to CD3^+^ T cells, there was little **j** CD4^+^-positive staining in the tissue or ventricles of age-matched wild-type littermates. Arrowheads indicate CD4^+^ positive T cells. Scale bar represents 50 μm in the hippocampus and ventricle images; inset scale bar represents 20 μm.

Blood component infiltration into the brains of aged rTg4510 mice suggested impaired BBB integrity. Therefore, we sought to determine if whole peripheral blood cells could also enter the parenchyma with age. Brain sections from 6-, 9-, and 12-month old rTg4510 mice were stained with hematoxylin and eosin (H&E) to visualize red blood cells (RBCs; erythrocytes) that remained in the brain parenchyma following perfusion [51]. RBCs, which are highly eosinophilic, stain bright red with circular morphology void of blue nuclear staining by H&E staining. These RBCs were manually counted throughout at least 6 sections from each mouse in the 6-, 9-, and 12-month old cohorts. Significantly more RBCs were found in the rTg4510 parenchyma at 12 months of age (Figure 6a, b). RBCs appeared to infiltrate the tissue near ventricles, the outer edges of the cortex, and along vasculature, particularly in the hippocampus.

As BBB defects are often correlated with entry of immune cells into the brain, staining for CD3^+^ T cells was performed. CD3^+^ T cells significantly infiltrated the brain of 12-month old rTg4510 mice (Figure 6c), especially near the longitudinal hippocampal blood vessels (internal transverse hippocampal artery and/or vein; Figure 6d) and periventricular to the lateral ventricles (Figure 6e), but were rarely observed in age-matched wild-type mice (Figure 6f). CD4^+^ T cells were also found to significantly infiltrate the brains of 12-month old rTg4510 (Figure 6g) mice compared to their younger counterparts and to age-matched wild-type mice. The infiltration of these CD4^+^ T cells were found in similar regions as CD3^+^ T cells, along the longitudinal hippocampal blood vessels (Figure 6h) and near periventricular regions (Figure 6i and j). Taken together, these data show that BBB disruption in 12-month old rTg4510 mice is sufficient to not only allow blood components into the brain, but also whole blood cells.

### Tau suppression by doxycycline restores BBB integrity

**Figure 7:**
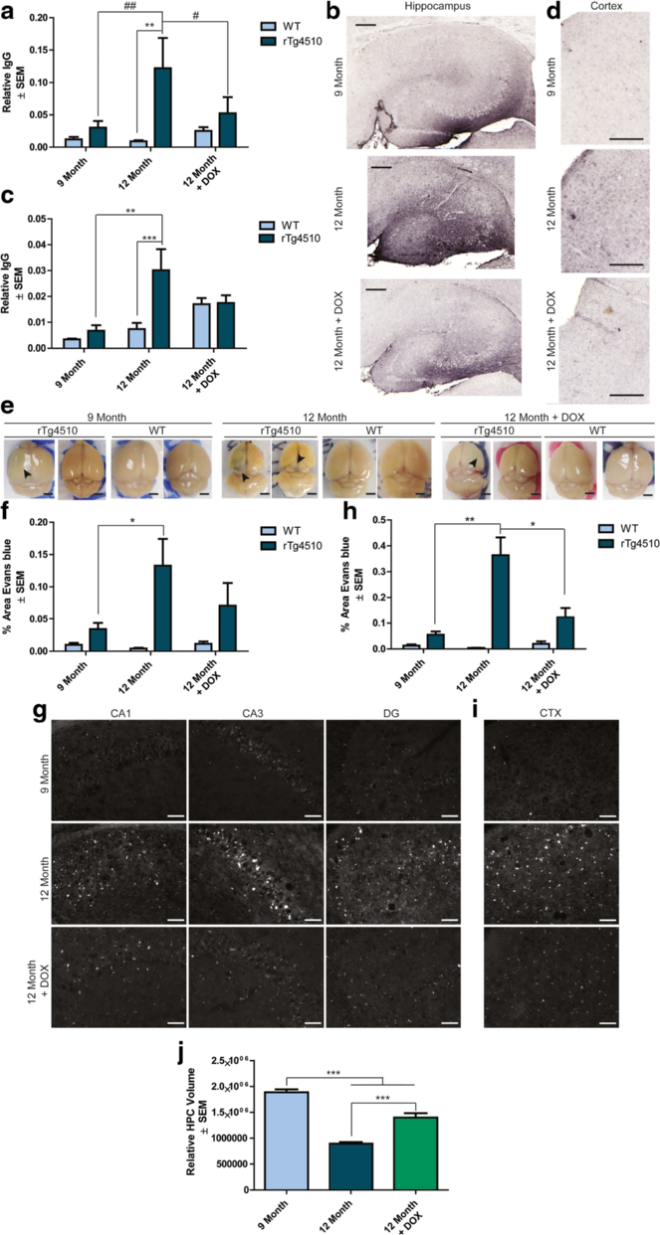
**Tau suppression by DOX slows BBB defect in 12-month old rTg4510 mice.** IgG levels in the **(a and b)** hippocampus (± SEM) and **(c and d)** frontal cortex (± SEM) of 12-month old DOX-treated mice compared to 9- and 12-month old wild-type and rTg4510 mice; Representative images are shown. *** *p* <0.001, ** *p* <0.01. Scale bar represents 200 μm. Images of **e** whole brains immediately following perfusion to observe Evans blue (EB) (*black arrowhead*) in 12 month DOX-treated mice compared to 9 and 12 month old rTg4510 relative to matching wild-type mice. Scale bar represents 200 μm. Positive EB expression was measured in the **(f and g)** hippocampus (± SEM) and **(h and i)** frontal cortex (± SEM) of 12 month old DOX-treated mice compared to 9- and 12-month old rTg4510 and age-matched wild-type mice; representative images of 9-month old, 12-month old, and DOX-treated 12-month old mice are shown. * *p* <0.05**, *p* <0.01. Scale bar represents 50 μm. Relative **j** hippocampal brain volume (± SEM) in 9- and 12-month old rTg4510 mice compared to 12-month old rTg4510 mice treated with DOX. *** *p* <0.001.

We next sought to test the resiliency of the BBB to damage caused by tau accumulation. We took advantage of the ability to suppress tau expression in the rTg4510 model using DOX feed. DOX was fed to 10.5- and 11-month old rTg4510 and wild-type mice for ~5 weeks. At 12 months of age, mice were injected with EB forty minutes before perfusion and tissue was collected following this in an identical manner to the cohorts described above. Immunostaining revealed that the levels of IgG were lower in 12-month old rTg4510 mice that received DOX treatment compared to the untreated group. In fact, the IgG immunoreactivity in the hippocampus (Figure 7a and b) and frontal cortex (Figure 7c and d) of mice treated with DOX beginning at ~11 months was indistinguishable from 9-month old rTg4510 mice, suggesting that tau suppression by DOX could slow and possibly reverse the BBB dysfunction in these mice. Superficial examination of EB staining also suggested that DOX-treated 12-month old rTg4510 mice resembled 9-month old rTg4510 mice with regard to EB extravasation (Figure 7e). Histochemical analysis of the area ratio of EB extravasation in these mice showed that the hippocampi of 12-month old DOX-treated rTg4510 mice were quantitatively indistinguishable from either 9-month old or 12-month old untreated rTg4510 (Figure 7f and g), and the frontal cortex had significantly lower levels of EB compared to 12-month old untreated rTg4510 mice (Figure 7h and i). Similar trends were observed for EB particle count, total area positive and average particle size (Additional file 1: Figure S3) Taken together, this suggests that BBB integrity was maintained and in part restored following tau suppression by DOX. As expected, hippocampal atrophy was also significantly slowed by DOX treatment, as measured by traces used for segmentation from NearCYTE software (Figure 7j). However, 12-month old DOX-treated rTg4510 mice still had significantly lower hippocampal volume than 9-month old untreated rTg4510 mice. Altogether, these data suggest that tau suppression can slow and possibly even rescue the progression of BBB dysfunction in this model of tauopathy.

### Tau suppression slows and possibly reverses BBB dysfunction in aged rTg4510 mice

**Figure 8:**
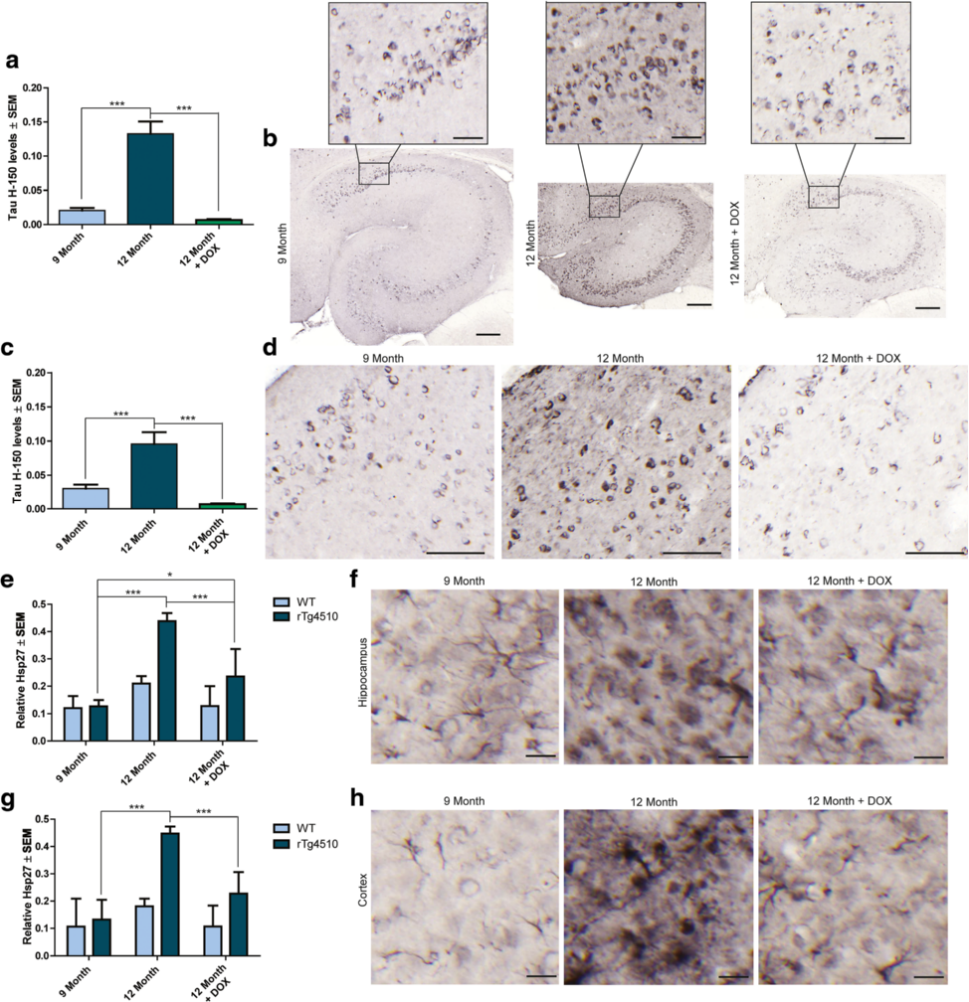
**Effects of tau suppression on tau and Hsp27 levels.** Quantification of relative tau (H-150) levels in the **(a and b)** hippocampus (± SEM) and **(c and d)** frontal cortex (± SEM) of DOX-treated 12-month old rTg4510 mice compared to 9- and 12-month old untreated rTg4510 mice; representative images are shown. Scale bar in cortical images represents 100 μm. *** *p* <0.001 Scale bar in hippocampal images represents 200 μm; inset scale bar represents 50 μm. Relative levels of Hsp27 in the **(e and f)** hippocampus (± SEM) and **(g and h)** frontal cortex (± SEM) from 12-month old DOX-treated mice compared to 9- and 12-month old mice, relative to wild-type controls; representative images are shown. Scale bar represents 20 μm. ** *p* <0.01, *** *p* <0.001.

As expected, total tau levels in DOX-treated mice were significantly decreased in the hippocampus (Figure 8a and b) and frontal cortex (Figure 8c and d) compared to untreated rTg4510 mice. As depicted by the representative images in the hippocampus (Figure 8b) and frontal cortex (Figure 8d), there was a noticeable reduction of the dispersed tau-positive staining in the 12-month old DOX-treated and 9-month old rTg4510 mice compared to 12-month old untreated rTg4510 mice. In fact, the DOX treated mice were statistically indistinguishable from 9-month old untreated rTg4510 mice. This reduction in dispersed tau staining may be due to a decrease in more soluble tau species which are lowered by DOX, since previous studies showed that insoluble neurofibrillary tangles are not cleared by tau suppression [23]. Hsp27 accumulation was also significantly reduced by tau suppression in both the hippocampus (Figure 8e and f) and frontal cortex (Figure 8g and h), but was still significantly higher than 9-month old untreated rTg4510 mice in the hippocampus (Figure 8e). In addition to slowing the accumulation of the levels of Hsp27, the morphology of cells expressing Hsp27 appears to be slightly altered in both the hippocampus (Figure 8f) and frontal cortex (Figure 8h), suggesting that distinct activation states of astrocytes may also contribute to BBB disruption.

**Figure 9:**
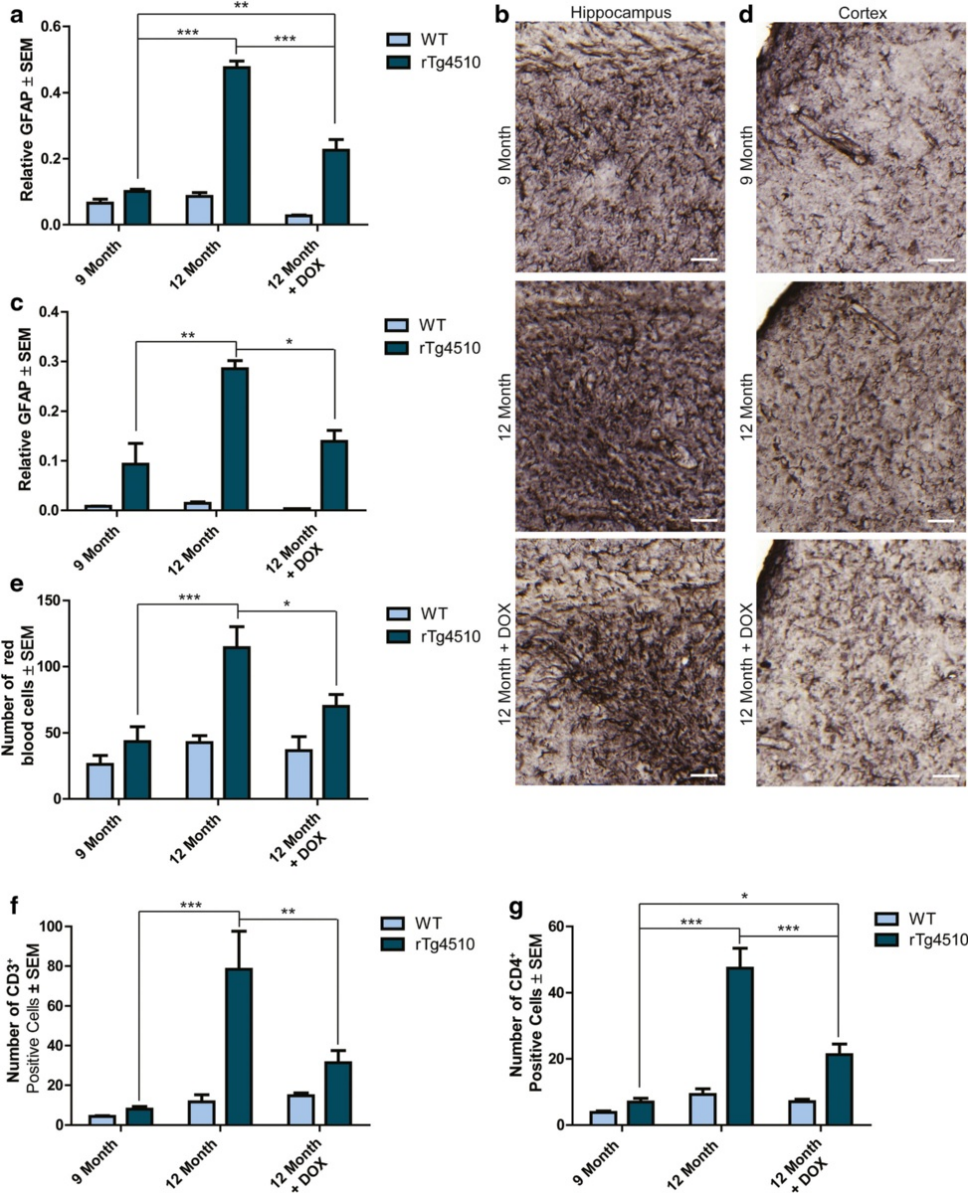
**Effects of tau suppression on GFAP levels, RBCs and infiltration of T cell lymphocytes.** Relative levels of GFAP positive astrocytes in the **(a and b)** hippocampus and **(c and d)** frontal cortex of 9-, 12-, and 12-month old + DOX treated rTg4510 and wild-type mice; representative images are shown. Scale bars represent 50 μm. * *p* <0.05, ** *p* <0.01, *** *p* <0.001. Number of **e** RBCs (± SEM) counted from H&E staining of 12-month old DOX-treated and 9-and 12-month old untreated rTg4510 mice relative to the levels of their wild-type littermates. * *p* <0.05, *** *p* <0.001. Number of **f** CD3^+^ (± SEM) and **g** CD4^+^ (± SEM) T cell lymphocytes in 9-, 12- and DOX-treated 12-month old rTg4510 mice compared wild-type controls * *p* <0.05, ** *p* <0.01, *** *p* <0.001.

Confirmation of the reduction of activated astrocytes in DOX-treated rTg4510 mice was verified by GFAP staining, which was recently shown to be rescued by DOX in a separate study [43]. Consistent with Hsp27 staining, GFAP immunoreactivity was significantly lower in the hippocampi of 12-month old DOX-treated rTg4510 mice compared to their untreated counterparts (Figure 9a and b), but still significantly higher than 9-month old untreated rTg4510 mice. In the frontal cortex, GFAP immunoreactivity in 12-month old DOX-treated mice was significantly lower than 12-month old untreated rTg4510 mice, instead resembling 9-month old rTg4510 mice (Figure 9c and d).

T cell lymphocyte and RBC infiltration were also reversed by tau suppression. The RBC counts from 12-month old DOX-treated rTg4510 mice was significantly lower than 12-month old untreated rTg4510 mice and were indistinguishable from 9-month old untreated rTg4510 mice (Figure 9e). We also found that CD3^+^ (Figure 9f) and CD4^+^ (Figure 9g) T cells were significantly lower in the DOX-treated rTg4510 mice compared to untreated mice. Unlike CD3^+^ T cells, CD4^+^ T cells, while reduced, were still significantly higher in 12-month old DOX-treated rTg4510 mice compared to 9-month old rTg4510 mice.

### Appearance of perivascular tau in 12-month old rTg4510 mice

**Figure 10:**
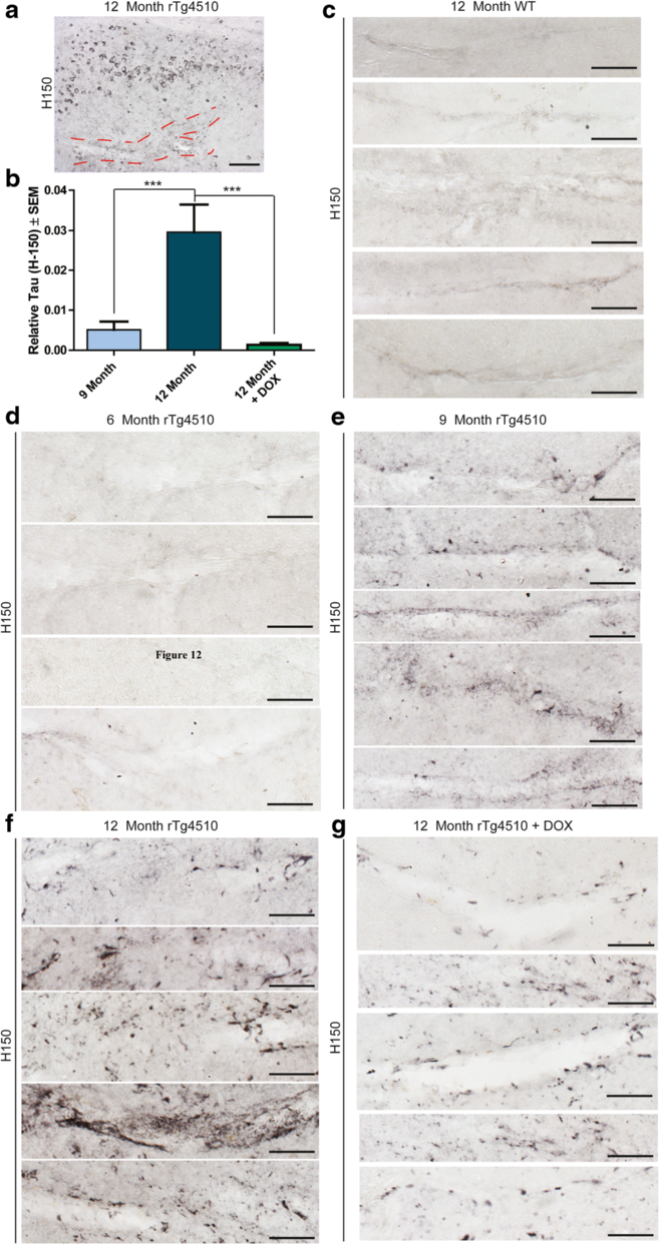
**Tau accumulates along hippocampal blood vessels in 12-month old rT4510 mice.** 20x magnification image of the **a** hippocampus of a 12-month old rTg4510 mouse stained with tau (H-150) to show the region of perivascular tau accumulation. Scale bar represents 100 μm. Relative levels of **b** tau (H-150) (± SEM) were measured in and around the longitudinal hippocampal blood vessels in 9- and 12-month old and 12-month old rTg4510 mice treated with DOX. ***p <0.001. Representative images of tau (H-150) stained tissue from a **c** 12-month wild-type mouse and rTg4510 mice at **d** 6, **e** 9, and **f** 12 months of age, and **g** 12-month old rTg4510 mice DOX-treated mice are shown. Scale bars represent 50 μm.

**Figure 11:**
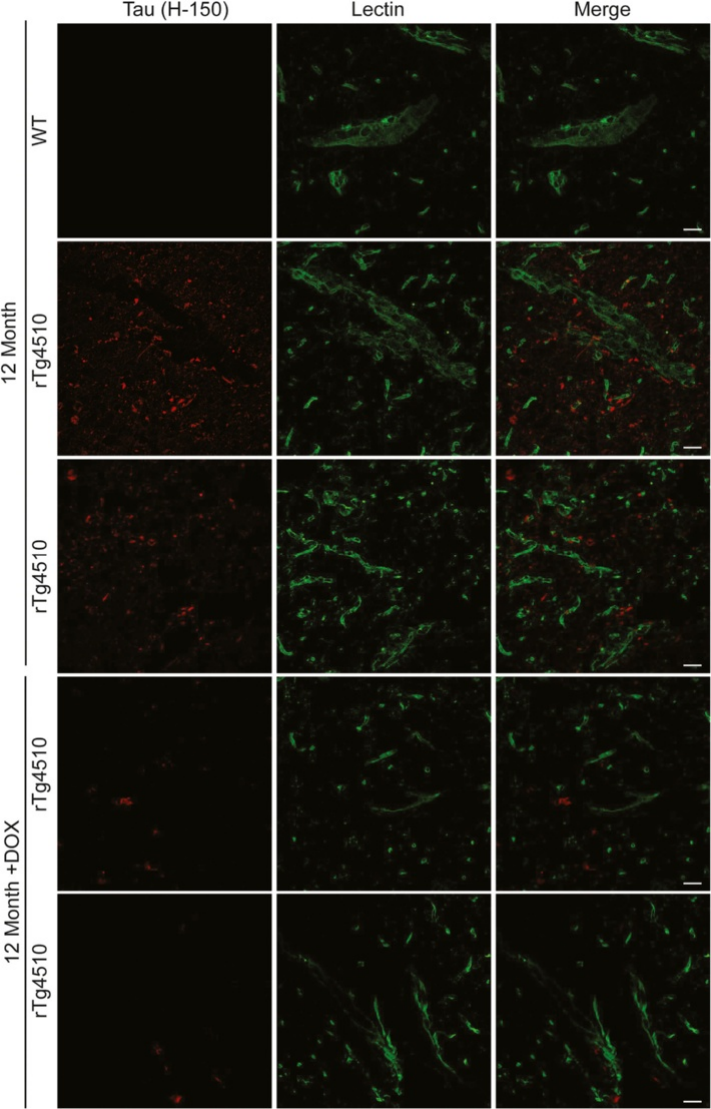
**Perivascular tau is found along the blood vessels in 12-month old rTg4510 mice.** 63x magnification images of tau (H-150; red) and Lectin 488 (green) along the hippocampal blood vessels from 12-month old WT, rTg4510 and DOX-treated mice. Scale bar represents 20 μm.

Since the hippocampi of the rTg4510 model develop the most robust and aggressive tau pathology, we examined this area for any apparent differences in the morphology of tau staining. Through this process, we discovered tau immunoreactivity in and around the longitudinal blood vessels of the hippocampus (Figure 10a; additional examples are included in Additional file 1: Figure S4a) [52,53]. Quantification of this perivascular tau revealed that it was significantly more abundant in 12-month old rTg4510 mice than in 9-month old rTg4510 mice or 12-month old DOX-treated rTg4510 mice (Figure 10b). Tau was not localized to this region in 12-month old wild-type mice (Figure 10c) or in 6-month old rTg4510 mice (Figure 10d). It only emerged in rTg4510 mice 9 months of age or older (Figure 10e). We found that the accumulation of tau in and around these blood vessels was significant by 12-months of age (Figure 10f), but was markedly reduced in DOX-treated rTg4510 mice (Figure 10g). Lectin and tau co-staining (Figure 11) revealed that tau was found closely lining the endothelial cells of the blood vessels in the hippocampus in 12-month old rTg4510 mice. This was not found in age-matched WT mice and appeared less robust in 12-month old DOX-treated mice. These findings suggest that tau along these vessels is not within endothelial cells based on the lack of co-localization of tau with lectin staining. We then performed co-localization studies of Hsp27, tau and EB to better define the location of this perivascular tau. Tau was found co-localized with both EB and Hsp27, suggesting that tau and EB were within Hsp27 positive cells along the vasculature (Additional file 1: Figure S4b). Since tau did not appear to co-localize with endothelial cells, and since tau has not been found in neuroglia up to 13 months in the rTg4510 mice by electron microscopy [54], we can conclude that the perivascular tau is likely either within neuronal processes that could contain Hsp27 and EB, or it could be extracellular as both EB and Hsp27 are found outside of cells as well [55,56]. Regardless of the localization of this perivascular tau, its emergence coincides much more closely with BBB dysfunction than other pathologies in this model, such as neuronal loss and gliosis. Thus, these findings suggest an important relationship between this perivascular tau pathology and BBB integrity.

## Discussion

**Figure 12:**
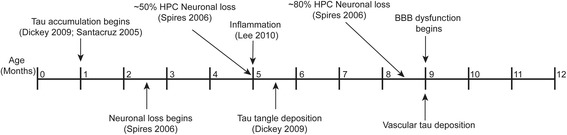
**BBB breakdown correlates best with perivascular tau levels.** Representative schematic depicting the timeline of progressive pathologies found in rTg4510 mice including tau accumulation, neuronal loss, tau tangle deposition, inflammation, appearance of perivascular tau, and initiation of increased BBB permeability. Tau accumulation begins at a very young age, before one month, followed by regional neuronal loss. A few months after tau tangles start to deposit just after signs of inflammation are noticeable. Neuron loss continues to get more severe with age and by 9 months vascular tau is detectable in the hippocampus, concomitant with the start of increased BBB permeability.

This study is the first to demonstrate that tau derived from neurons can be sufficient to initiate BBB impairment. This dysfunction was found to be concomitant with perivascular tau accumulation, subsequent to the onset of other pathologies found in this mouse model. In fact, the BBB is remarkably stable in the face of robust tau accumulation, neuroinflammation, and neurodegeneration. This is based on our data showing that BBB dysfunction only manifested once vascular tau emerged at 9 months, well after neuronal loss and total tau accumulation begin at 2.5 months and gliosis at 4 months (Figure 12) [24,43]. In fact, BBB function in the rTg4510 mice was found to deteriorate at a similar time point as the emergence of perivascular tau along the hippocampal vessels (Figure 10f, g and h). The accumulation of tau in the forebrain and hippocampus [24] is the nexus which leads to BBB dysfunction in the rTg4510 model, either directly or indirectly.

Tau-induced neuronal loss and inflammation are apparent much earlier in the rTg4510 model than BBB dysfunction. But while inflammation in particular is a known regulator of BBB stability [57], gliosis emerges at nearly the same time and place as tau aggregates begin to appear in the rTg4510 model, both of which occur at a much younger age relative to the emergence of BBB pathology. This suggests that there may be a threshold that tau-induced inflammation and/or neurodegeneration must surpass to induce BBB permeability at 9 months in this model. Alternatively, it could be a distinct pathology that arises, such as the accumulation of perivascular tau, or a combination of these factors. Interestingly, BBB damage in this model appeared greatest in areas such as CA3 and the surface of the cortex, adjacent to the areas that have the most robust neuronal loss and gliosis such as CA1 and frontal mid-cortical layers. In fact, BBB damage was highest near the ventricles and the meninges, the same areas that show signs of BBB leakage in normal aging [37]. In this way, the BBB damage in the rTg4510 brain is similar to that in the normal aging brain with regard to location of initial insult; however this pathology is greatly accelerated, suggesting that the pathologies caused by tau accumulation somehow interact with normal aging pathologies to cause this BBB damage. While the precise mechanism contributing to BBB dysfunction in these mice remains unknown, it is clear that the brain is highly resistant to BBB damage in the face of major pathological insults, and only results through a complex pathogenic cascade much later in the rTg4510 brain.

Still, the specific mechanism by which tau causes BBB dysfunction remains unclear. Perhaps tau aggregation works in a way that is similar to vascular Aß, by decreasing glucose transporters [58] stimulating reactive oxygen species [59], and increasing inflammatory molecules [59-61]. In fact, tau accumulation is associated with increased inflammation leading to stimulation of TNF-α and MCP-1, and both of these have been implicated in BBB dysfunction [21,62]. Moreover, the tau accumulating along the vasculature in the rTg4510 mice could be extracellular, perhaps suggesting some type of physical damage to the endothelial cells. There is evidence that tau transgenic models do produce extracellular tau [63,64] and recent work suggests that tau can spread in a prion-like manner in these same mouse models [65-67], further suggesting the potential for a pathological role of extracellular tau. Tau has also been shown to accumulate into annular protofibrils, which may be able to puncture membranes [68], suggesting perhaps another way for tau to damage the BBB. It is also possible that tau is able to damage the BBB from within cells. In human AD brain, it was recently demonstrated that tau is not only found near the vasculature [14], but tau oligomers specifically co-localized with endothelial cells within the blood vessels, suggesting that tau was either extracellular or within pericytes or endothelial cells [69]. However our data would suggest that this is not the case in the rTg4510 model, since tau did not strongly co-localize with lectin staining. Another possibility is that neuronal tau accumulation triggers astrocytosis, causing these cells to detach from tight junctions. Also, while likely not relevant to this model because there is no pathological evidence of glial tau in the rTg4510 mice, astrocytic tau has been shown to compromise the BBB by damaging endfeet [18]. Still another mechanism is that tau within endothelial cells themselves can become aberrantly phosphorylated, leading to BBB disruption [54,70,71].

Interestingly, vascular tau is also found in AD cases with CAA [72-74], but this has typically been hypothesized to be downstream of Aß-triggered BBB dysfunction, rather than a causal pathology. There is also evidence that mouse models expressing both mutant amyloid precursor protein or Presenilin 1 have some tau present along the vasculature [13,75], but this was speculated to be a consequence of Aß deposition rather than a pathophysiological function of tau. Thus, while the prevailing dogma in AD is that Aß triggers the BBB dysfunction, our findings clearly show that tau alone is capable initiating impaired BBB stability independent of Aß.

Perivascular tau is also present in progressive supranuclear palsy (PSP) and corticobasal degeneration (CBD), suggesting that AD is not the only tauopathy to show a BBB defect [76,77]. In contrast to PSP and CBD, AD tau tangles contain exon 10 (+) and exon 10 (-) tau species while tangles from PSP and CBD are exclusively exon 10 (+). Both PSP and AD have neuritic plaques, but only AD has neuropil threads. Moreover, tau pathology in the AD brain originates in the entorhinal cortex and hippocampus, eventually spreading throughout the forebrain, but in PSP tau pathology is restricted to the basal ganglia, brainstem, substantia nigra, and subthalamic nucleus [78,79], and in CBD it is restricted to the cerebral cortex [79]. Perhaps most distinct is that tau in PSP and CBD brain accumulates within astrocytes [76], while AD brain shows no signs of neuroglial tau pathology. Despite these distinct pathologies, like AD, there is clear evidence for BBB damage in PSP that has been associated with decreased P-glycoprotein, a transporter found in endothelial cells within the BBB [15,77]. Unlike AD where the perivascular tau is thought to arise from neurons, perivascular tau in PSP was attributed to tau derived from tangle bearing thorn-shaped glial cells [80] and perivascular tau in CBD is most closely associated with astrocytic tau [81]. But, despite different origins, perivascular tau accumulates in each of these diseases, providing further support for a role of tau pathology in BBB damage. Thus, the rTg4510 mice likely most accurately model the BBB defect caused by tau in the AD brain since the tau is neuronally derived and the location of the pathology. However, since the accumulation of perivascular tau is common in each disease, it is possible that the rTg4510 mice, which also have perivascular tau accumulation, could define the role of this particular tau pathology in BBB function.

## Conclusion

In summary, we show that tau derived from neurons causes a progressive BBB dysfunction that is most closely correlated with the emergence of perivascular tau, rather than the robust neuronal loss and neuroinflammation that occurs much earlier in the pathogenic cascade of the rTg4510 model. Importantly, reducing tau levels recovered the BBB defect, demonstrating its remarkable resiliency, even in the face of dramatic degeneration and inflammation. Thus, the threshold for BBB integrity is extremely high in the mouse brain and as a result BBB damage appears to be the last chip to fall in the neurotoxic sequelae of this model. Furthermore, this BBB defect in the rTg4510 mouse model can now be used to more adequately model the pharmacokinetic profile of promising pre-clinical therapies to treat tauopathies, since BBB dysfunction is found in AD, traumatic brain injury (TBI) and other tauopathies. Since peripheral infiltration of inflammatory components into the brain is thought to contribute to the late stage sequelae in AD and other tauopathies [82-85], our findings suggest that even late stage interventions targeting tau could help maintain BBB integrity and reduce the vascular contributions to cognitive impairment and dementia that occur in these diseases. Overall, this work demonstrates that tau accumulation can damage the BBB to the point that whole peripheral cells can now enter the brain, perhaps initiating a secondary disease cascade involving the peripheral immune system. But this process is late in the disease process and can be stopped if tau levels are reduced.

## Additional file

Additional file 1: Figure S1. Evans blue fluorescence in subregions of the hippocampus. Quantification of EB fluorescence in the CA1, CA3, and dentate gyrus regions of the hippocmapus from 12-month old rTg4510 and wild-type mice. ***p<0.001. **Figure S2.** Hippocampal atrophy with aging in the rTg4510 mouse model. Quantification of relative hippocampal volume at 6-, 9- and 12-months. *p<0.05, ***p<0.001. **Figure S3.** Additional Evans blue (EB) measurements. Average count of independent EB positive spots per **a** hippocampus and **b** frontal cortex. *p<0.05, **p<0.01. Total EB positivity (pixels) in each **c** hippocampus and **d** frontal cortex. ***p<0.001. Average size of independent EB positive areas per **e** hippocampus and **f** frontal cortex. ***p<0.001. **Figure S4.** Perivascular tau is found along the hippocampal blood vessels. **a** Total tau (H-150) reactivity in the hippocampus of 12–month old rTg4510 mice. Images taken at 20× magnification. Scale bar represents 100 μm. **b** Representative images of hippocampal blood vessels from 12-month old wild-type, rTg4510 and rTg4510 + DOX stained for Hsp27, tau (v-20), and Evans blue. Images taken at 60x magnification. Scale bar represents 50 μm.

## Abbreviations

Aβ: Amyloid beta
AD: Alzheimer’s disease
BBB: Blood-brain barrier
CAA: Cerebral amyloid angiopathy
CBD: Corticobasal degeneration
DOX: Doxycycline
EB: Evans blue
GFAP: Glial fibrillary acidic protein
H&E: Hematoxylin and eosin
Hsp27: Heat shock protein 27 kDa
NFT: Neurofibrillary tangle
PSP: Progressive supranuclear palsy
RBC: Red blood cell
TBI: Traumatic brain injury
