# Supplementary material for: Tau depletion prevents progressive blood-brain barrier damage in a mouse model of tauopathy
Source: Acta Neuropathol Commun. 2015 Jan 31;3:8. doi: 10.1186/s40478-015-0186-2 (PMC4353464; doi:10.1186/s40478-015-0186-2)
Supplement: Additional file 1: Figure S1. — Evans blue fluorescence in subregions of the hippocampus. Quantification of EB fluorescence in the CA1, CA3, and dentate gyrus regions of the hippocmapus from 12-month old rTg4510 and wild-type mice. ***p<0.001. Figure S2. Hippocampal atrophy with aging in the rTg4510 mouse model. Quantification of relative hippocampal volume at 6-, 9- and 12-months. *p<0.05, ***p<0.001. Figure S3. Additional Evans blue (EB) measurements. Average count of independent EB positive spots per a hippocampus and b frontal cortex. *p<0.05, **p<0.01. Total EB positivity (pixels) in each c hippocampus and d frontal cortex. ***p<0.001. Average size of independent EB positive areas per e hippocampus and f frontal cortex. ***p<0.001. Figure S4. Perivascular tau is found along the hippocampal blood vessels. a Total tau (H-150) reactivity in the hippocampus of 12–month old rTg4510 mice. Images taken at 20× magnification. Scale bar represents 100 μm. b Representative images of hippocampal blood vessels from 12-month old wild-type, rTg4510 and rTg4510 + DOX stained for Hsp27, tau (v-20), and Evans blue. Images taken at 60x magnification. Scale bar represents 50 μm. [file 40478_2015_186_MOESM1_ESM.pdf]

Additional File 1

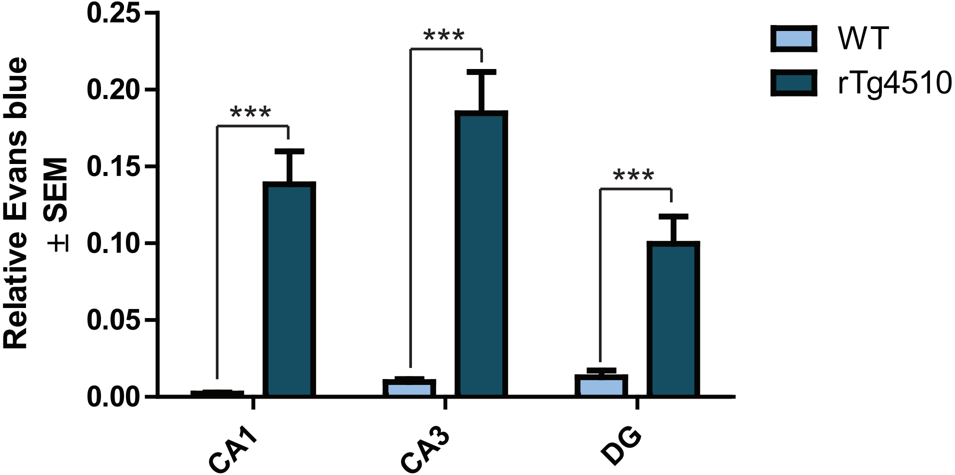

Figure S1 Evans blue fluorescence in subregions of the hippocampus. Quantification of EB fluorescence in the CA1, CA3, and dentate gyrus regions of the hippocmapus from 12-month old rTg4510 and wild-type mice. ****p*<0.001.

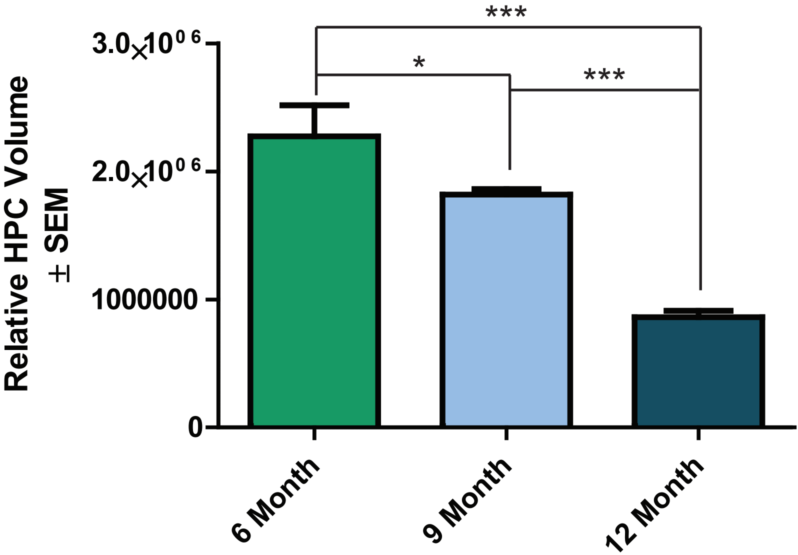

Figure S2 Hippocampal atrophy with aging in the rTg4510 mouse model. Quantification of relative hippocampal volume at 6-, 9- and 12-months. *p<0.05, ***p<0.001.

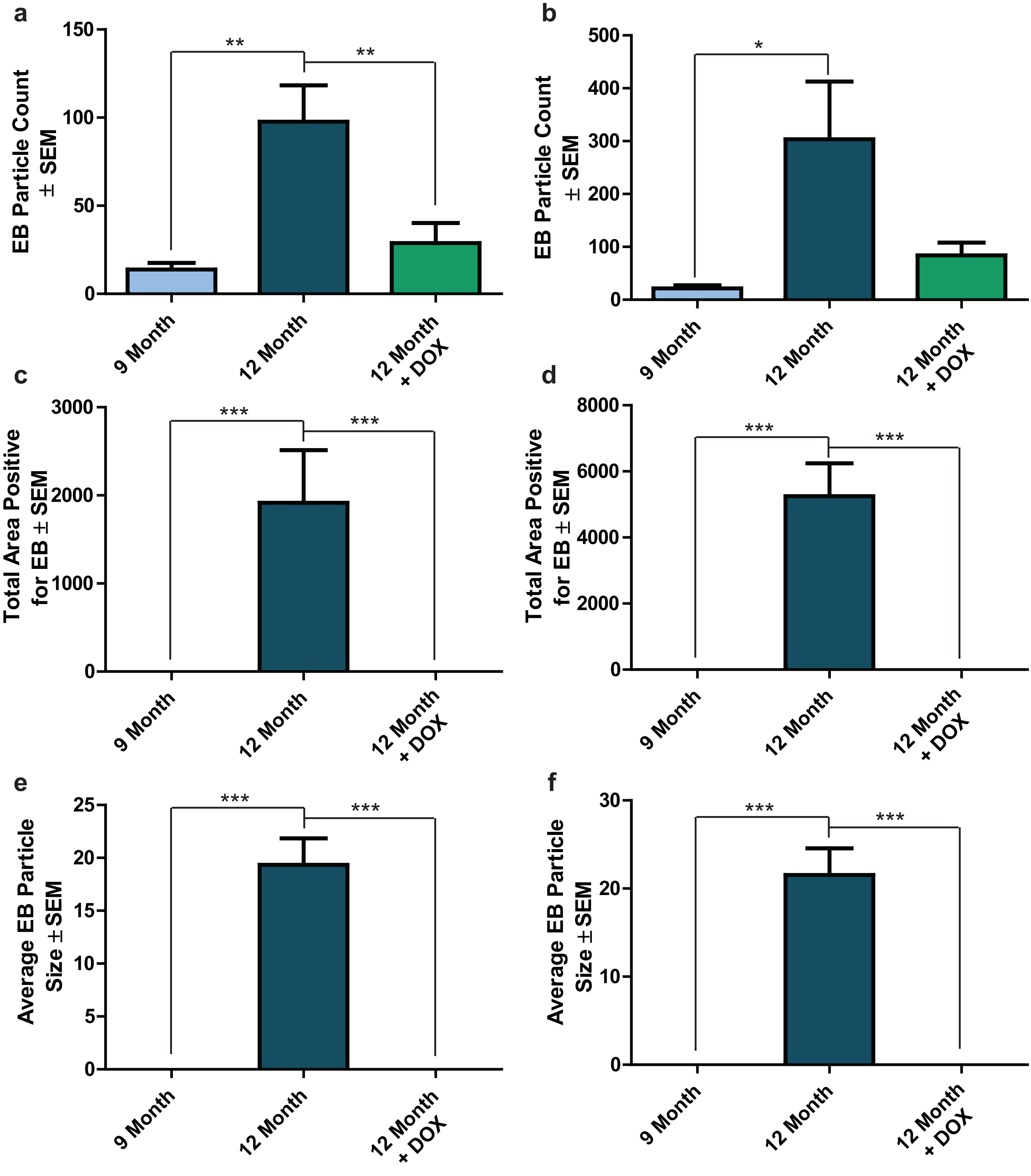

Figure S3 Additional Evans blue (EB) measurements. Average count of independent EB positive spots per **a** hippocampus and **b** frontal cortex. *$p<0.05$, **$p<0.01$. Total EB positivity (pixels) in each **c** hippocampus and **d** frontal cortex. ***$p<0.001$. Average size of independent EB positive areas per **e** hippocampus and **f** frontal cortex. ***$p<0.001$.

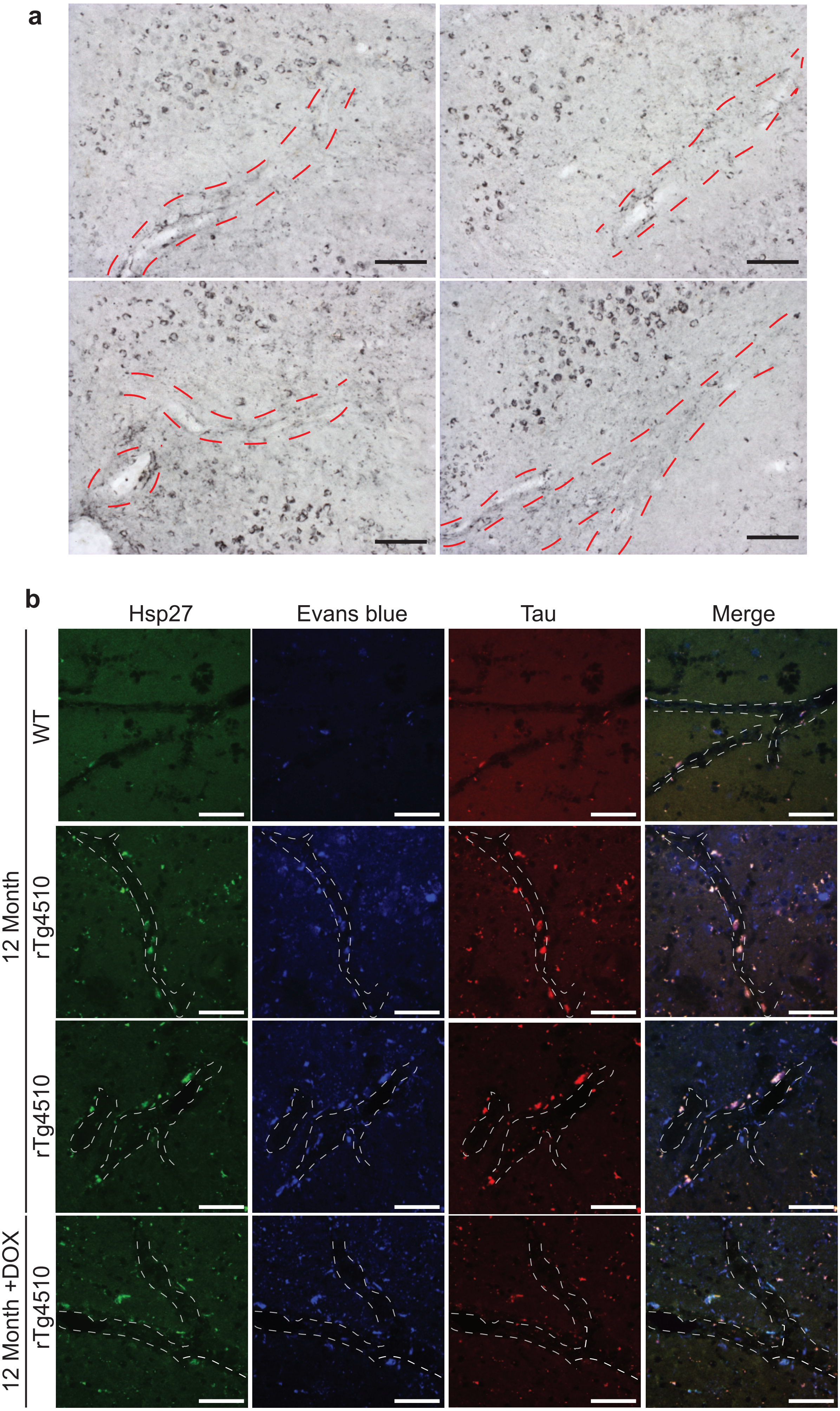

Figure S4 Perivascular tau is found along the hippocampal blood vessels. **a** Total tau (H-150) reactivity in the hippocampus of 12-month old rTg4510 mice. Images taken at 20x magnification. Scale bar represents 100 μm. **b** Representative images of hippocampal blood vessels from 12-month old wild-type, rTg4510 and rTg4510 + DOX stained for Hsp27, tau (v-20), and Evans blue. Images taken at 60x magnification. Scale bar represents 50 μm.
